# Hepatitis B Immune Globulin in Liver Transplantation Prophylaxis: An Update

**DOI:** 10.5812/hepatmon.832

**Published:** 2012-03-28

**Authors:** Payam Dindoost, Seyed Mohammad Jazayeri, Seyed Moayed Alavian

**Affiliations:** 1 Iranian Hepatitis Network, Tehran, IR Iran; 2 Hepatitis B Molecular Laboratory, Department of Virology, School of Public Health, Tehran University of Medical Sciences, Tehran, IR Iran; 3 Baqiyatallah Research Center for Gastroenterology and Liver Diseases, Baqiyatallah University of Medical Sciences, Tehran, IR Iran

**Keywords:** Liver Transplantation, Hepatitis B Hyperimmune Globulin, Prevention and Control

## Abstract

**Context:**

Liver transplantation is the best treatment option for end-stage liver disease following hepatitis B (HBV) infection. However, the high rate of recurrence of HBV infection following transplantation is a disadvantage of this option.

**Evidence Acquisition:**

Over the past 2 decades, the gold standard of prophylactic treatment for the prevention of HBV re-infection following liver transplantation has been the administration of low- to high-dose hepatitis B immune globulin (HBIg) along with an antiviral agent to induce passive immunity.

**Results:**

The effectiveness of HBIg in preventing the recurrence of HBV depends on the dosage, route of administration, and duration of HBIg treatment, and the viremic status at the time of transplantation. There is currently no consensus on a standardized recommendation for therapeutic options that include HBIg administration.

**Conclusion:**

This review attempts to summarize the available data on the feasibility of such options. Most recent studies support the use of long-term combination therapy of HBIg and antiviral NAs (especially new agents).

## 1. Context

Globally, 30% of cirrhosis cases and 53% of primary liver cancer cases are attributed to hepatitis B infection [1], for which liver transplantation (LT) has been the only standard treatment option [2]. However, the success of LT has been complicated by the high incidence (80%-100%) of recurrent graft infections, which typically occur between 6 and 12 months after LT in the absence of prophylaxis [3]. This is accompanied by subsequent graft failure, rapid progression to cirrhosis, fulminant hepatitis, and patient death. The frequency of HBV reinfection is the lowest in HBV-immune recipients (0%-18%) and is the highest in HBV-naive recipients (70%-80%) [2][4][5][6][7][8][9]. Reinfection has been hypothesized to occur due to the presence of circulating virions at the time of LT, exposure to extra-hepatic reservoirs of HBV, or the persistence of the HBV covalently closed circular DNA (cccDNA) as a replicative intermediate [8][10][11]. Furthermore, candidates under immunosuppressive therapy following LT commonly show a decrease in antibody titers [12] and an increase in viral replication [11][13]. Such active viral replication at the time of LT (> 10(5) copies/mL) was shown to be an important factor affecting HBV recurrence, sometimes regardless of immunoprophylaxis therapy [6][14][15]. Moreover, hepatitis B infection in LT recipients who have received grafts from hepatitis B core antibody positive donors is a serious medical concern [16]. Therefore, HBV-related liver disease was initially regarded as a contraindication to LT [17][18]. The morbidity and mortality caused by recurrent HBV infection after LT were significant before the advent of effective immunoprophylaxis [19]. However, in the last 2 decades, the indication for LT in patients with HBV-related end-stage liver disease has changed dramatically, and prophylactic strategies against HBV reinfection have changed outcomes for patients who undergo LT [20][21]. With adequate prophylaxis, less than 10% of the patients with HBV recurrence have to undergo repeated LT due to HBV-related graft failure [10][22][23].

## 2. Evidence Acquisition

The primary purpose of this review was three-fold. Our first aim was to clarify the effectiveness of different routes of administration, durations, and dosages of HBIg prophylactic options. The second aim was to compare 2 therapeutic options: HBIg monotherapy and HBIg therapy in conjunction with nucleos(t)ide analogues (NAs). Our third and final aim was to summarize the available studies on the different prophylaxis protocols used to prevent HBV recurrence in transplant recipients. A comprehensive search was performed in PubMed with the following Mesh search keywords: liver transplantation and HBIg monotherapy, liver transplantation and HBIg combination with NA, HBIg, and hepatitis B surface antigen (HBsAg) variants in liver transplantation. All published data from 1989 (the discovery of surface mutants) to August 2011 have been included in this review. Studies on the impact of HBIg monotherapy or HBIg therapy along with NAs on the HBV recurrence rate post-LT, HBIg route, dosage, and treatment, or mutations caused by the selective pressure following HBIg therapy were included in our analyses. Data were analyzed on the basis of sample size, rate of recurrence, and presence or absence of NAs in the different prophylactic protocols.

## 3. Results

### 3.1. HBIg and the Prophylactic Options

Over the past 2 decades, the gold standard of prophylactic treatment for the prevention of HBV reinfection following LT has been the inducement of passive immunity by administering low- to high-dose HBIg with or without antiviral NAs [10]. However, a consensus on the dose, duration, or route of administration of HBIg for the prevention and/or suppression of HBV reinfection post-LT is lacking, and protocols tend to vary widely from 1 transplant center to another [24][25][26][27]. However, with respect to the best practice for prophylactic strategies, most transplant programs in different centers around the world use the following 3 steps in general:

1) Intravenous administration of HBIg (4000-10,000 IU/mL) during the anhepatic phase.

2) Daily intramuscular or intravenous administration of 2000 to 10,000 IU HBIg for the first 3-7 days following LT.

3) High-dose therapy with 10,000 IU of HBIG every 4 weeks post-transplantation or low-dose therapy (400-2000 IU of HBIG) every 2 weeks post-transplantation to maintain anti-HBs > 100 IU/L [10][27].

### 3.2. Route of HBIg Administration

3.2.1. Intravenous Route

Traditionally, HBIg has been administered, pre-and postoperatively, at high doses, through the intravenous (IV) route. (Table 1) [4][5][15][19][25][28][29][30][31][32][33][34][35][36][37]. Despite the reported success of this method in preventing HBV recurrence post transplantation, highy costs and patient incompliance have limited the acceptance of this approach.

**Table 1:** The Different Studies With Different Strategies for Prevention of HBV Infection After Liver Transplantation

	**HBIg aRoute**	**HBIg Dose**	**HBIg Mono**	**HBV a Rec a, %**	**HBIg + LAM **a	**HBIg + New NA **a	**HBV Rec, %**	**Short Term HBIg**	**Indefinite HBIg**
Alonso et al. 2003 [85]	IM a	Low	X	16.6	-	-	-	-	X
Angus et al. 2008 [41]	IM	Low	X	0	X	ADF a	0		X
Angus et al. 2000 [40]	IM	Low			X		0		X
Anselmo et al. 2002 [14]	IM	Low	X	48	X		11		X
Akylidiz et al.2007 [39]	IM + IV a	Low			X	ADF	5.2		X
Buti et al. 2003 [73]	IM	Low	-	-	X	-	20	X	-
Compos-Varela et al. 2011 [38]	IM	Low	X	71	X	ENT a/TDF a/FTC^a^/ADF	7		X
Dadson et al. 2000 [86]	IM	Low	X	-	-	-	-	X	X
Faust et al. 2003 [87]	IM	Low	-	-	X	-	8.3		
Ferretti et al. 2004 [75]	IM	Low	-	-	X	-	8.3		X
Filipponi et al. 2010 [88]	IM	Low	X	0	X	-	0	X	-
Gane et al. 2007 [42]	IM	Low	-	-	X		4		X
Han et al. 2003 [72]	IM	Low	-	-	X		0	-	X
Jiang et al. 2010 [69]	IM	Low	-	-	X	ADV/ENT b	5.5	-	X
Jiao et al. 2007 [89]	IM	Low	-	-	X	-	5.4		X
Jiménez-Pérez et al. 2010 [80]	IM	Low	-	-	X	ENT + TDF	0		X
Karademir et al. 2006 [90]	IM	Low	-	-	X	-	0		X b
Lu et al. 2008 [45]	IM	Low	-		X		9	X	
Suehiro et al. 2005 [46]	IM	Low	-		X		0		X
Targhetta et al. 2006 [47]	IM	Low	X	16.6	X^b^	-	-		X
Takaki et al. 2007 [43]	IM	Low	-		X		0	X, 1y	
Umeda et al. 2006 [48]	NA b	Low	X	23.7	-	-	-		X
Woo et al. 2008 [44]	IM	Low	-		X	ADF^b^	4.2		X
Wang et al. 2004 [49]	IM	Low	-		X	ADF	3		X
Wong et al. 2007 [63]	IM	Low	-	-	X	ADF b	12.9	X	
Xi et al. 2009 [82]	IM	Low	-	-	X	ENT	11		X
Xie et al. 2010 [91]	IM	Low	-	-	X	ENT b	LAM:15.8; ENT:10		X
Yan et al. 2006 [2]	IM	Low	-		X		3.9		X
Yang et al. 2007 [92]	IM	Low	-	-	X	GAN a	4		X
Yilmaz et al. 2008 [51]	IM	Low	-		X	ADF	0		X
Yasunaka et al. 2011 [50]	NA	Low			X	ADF/ENT b	0 c		X
Zheng et al. 2006 [6]	IM	Low	-		X		16		X
Avolio et al. 2008 [28]	IV	High	X	30	X		9		X
Ben-Ari et al. 2003 [24]	IV	High	X	25	X	-	11		X
Chun et al. 2010 [93]	IV	High	X	13	X	-	10.2		X
Di Paolo et al. 2004 [74]	IV	High	-	-	X	-	0	X	-
Dickson et al. 2006 [29]	IV	High	-		X		3.3		X
Donataccio et al. 2006 [67]	IV	High	X	28.5	X b	ADF b	-	X	X
Dumortier et al. 2003 [30]	IV	High	X	23	X	-	0		X
Freshwater et al. 2008 [94]	IV	High	-	-	X	ADF	LAM: 62.5%; LAM + ADF: 0		X
Germer et al. 2003 [31]	IV	High	X	100	X^b^	X	0	-	-
Han et al. 2000 [19]	IV	High	X	25	X		0		X
Honaker et al. 2002 [52]	IV	High	X	21	X	-	0		X
Hwang et al. 2008 [32]	IV	High	X	6.4	X^b^	ADF/ENT b	-		X
Iacob et al. 2008 [33]	IV	High	-		X		4.8		X
Kwon et al. 2006 [34]	IV	High	X	0	X^b^		15		X
Lee et al. 2000 [95]	IV	High	X	0	X		0		X
Lee et al. 2004 [35]	IV	High	X	21.4	-	-	-		X
Marzano et al. 2001 [59]	IV	High	-	-	X	-	3	-	X
Marzano et al. 2005 [15]	IV	High	X	9	X	ADF	8		X
Manzarbeitia et al. 2002 [36]	IV	High	X	8.3	-	-	-	X, 6 mo	
Neff et al. 2004 [25]	IV	High	-	-	X	ADF/TDF b	12	X	
Roche et al. 2010 [4]	IV	High	X	17.7	X	ADF/TDF/ENT/FTC	0		X
Roque-Afonso et al. 2002 [5]	IV	High	X	6.2	-	-	-		X
Steinmuller et al. 2002 [37]	IV	High	X	16	X	FAM a	3.8		X
Anderson et al. 2007 [96]	IV	High	-	-	X	-	19.5		X
	IM	Low	-	-	X	-	5.9		X
Singham et al. 2010 [56]	SC a	Low	X	0	NO		-	X	
Yi et al. 2007 [97]	IV	High	X	6.3	X	-	13.8		X
Yoshida et al. 2007 [98]	IV	High	-	-	X	-	4	X	

^a^ Abbreviations: ADF, adefovir; ENT, entecavir; FAM, famciclovir; FTC, emtricitabine; GAN, ganciclovir; HBIg, hepatitis B immune globulin; HBV, hepatitis B virus; IM, intramuscular; IV, intravenous; LAM, lamivudine; LT, liver transplantation; NA, nucleot(s)ide analogous; Rec, recurrence; SC, subcutaneous; TDF, tenofovir

^b^ In the case of recurrence

^c^ HBsAg negative but intrahepatic cccDNA positive

3.2.2. Intramuscular Route

The administration of low dose intramuscular (IM) HBIg has been discussed as an accepted alternative route to IV administration in several studies worldwide (Table 1)[2][3][14][38][39][40][41][42][43][44][45][46][47][48][49][50][51][52]. However, given the limited volumes that can be given per IM injection, only patients with low HBIg requirements are suited for IM HBIg administration [9][53]. A crossover study employed alternative IM and IV regimens; in that study, intraindividual variation in antibody levels following IM and those following IV administration of HBIg were observed, but the overall anti-HB antibodies concentrations and half-lives in different individuals were not significantly different [54].

3.2.3. Subcutaneous Route

Two studies on the subcutaneous (SC) administration of HBIg, either alone or in combination with lamivudine (LAM), to prevent recurrent HBV infection in patients who had chronic hepatitis B before LT showed that SC administration of HBIg can effectively maintain satisfactory levels of anti-HB antibodies (Table 1). Both studies suggested SC delivery of HBIg as an alternative when IM or IV dosing are not possible [55][56]. The benefits of SC administration of HBIg include the potential for self-administration by patients, lower dosage (500-1000 IU), few adverse drug effects, stability of anti-HB levels, and an especially low recurrence rate [21][54][55][56].

### 3.3. HBIg Dose

3.3.1. High Dose HBIg Regimen

Numerous reports from different centers have shown that the continuous use of high-dose HBIg post-LT dramatically reduces the incidence of recurrent HBV infections. The high dose regimen was pioneered by Samuel et al., who showed that HBV infection could be reduced in frequency by administration of high doses of HBIg for more than 6 months after LT [20]. Later, 2 studies from the United States used aggressively high doses of IV HBIg, and subsequently, the recurrence rate reduced by more than 20% [57][58]. Thus, long-term, high-dose HBIg became the standard prophylaxis for HBV reinfection in most transplant centers in the 1990s, prior to the availability of effective antiviral therapies [11]. Further studies indicated that HBV reinfection could be prevented in majority of patients by using high-dose HBIg protocols and subsequent close monitoring of anti-HB antibody titers to prevent prophylaxis failures, especially in patients with high HBV DNA pre-LT (Table 1) [4][5][15][19][25][28][29][30][31][32][33][34][35][36][37][52]. Despite monitoring, patients with active replication still had a 16% rate of HBV recurrence. Subsequently, outcomes of a combination of high-dose IV HBIg and LAM were extensively investigated, and several studies reported a lower HBV recurrence rate (< 10% at 1- to 2-year follow-up) with this combination treatment (Table 1) [19][21][40][59][60]. However, the application of long-term high dose HBIg has been associated with high costs, inconvenience to patients, and the emergence of surface Ag mutations [4][6][10][53][61][62].

3.3.2. Low Dose HBIg Regimen

Some reports have shown that the efficacy of the low-dose HBIg and LAM combination therapy is similar to that of the high-dose protocol (Table 1) [41][42][47]. In several studies, administration of HBIg at low doses (300-800 IU) through the IM rather than the IV route has been shown to reduce the cost of prophylactic therapy, making the low-dose protocol as effective as the high-dose protocol [7][8][14][19][38][39][41][42][43][44][45][46][47][48][49][50][51][59][63] (Table 1). As a result of the combination with lamivudine, this approach was associated with similar recurrence rate and survival [40][42] that could effectively prevent HBV recurrence after liver transplantation [2]. Further, pre-LT reduction in viral replication makes the above combination therapy more effective [20][40]. Clearly, lower doses of HBIg (300-800 IU) and administration through the IM rather than the IV route reduce the cost of prophylactic therapy [8][14][19][40][42][59]. Currently, in several transplant centers in the United States, use of low doses of HBIg and a more limited duration of therapy have been shown to be sufficient in preventing HBV recurrence, as long as the appropriate therapy is started [64].

### 3.4. Indefinite Versus Short-Term Approaches

Indefinite HBIg prophylaxis may be required for patients with active replication at the time of initiation of LAM treatment [29]. Lifelong prophylaxis by the administration of HBIg in combination with NAs has resulted in a significant reduction in recurrent hepatitis B (Table 1) [10][17][37][65]. Currently, this approach is being employed in many transplant centers in the United States, despite a lack of significant differences between the different HBIg regimens in terms of HBV recurrence 5 years after LT [65]. Although compared to other treatments, indefinite, high dose HBIg treatment combined with NAs is thought to be very effective, the cost of this treatment is very high and as a result, in recent years, several centers have preferred to use short-term HBIg treatment in combination with indefinite nucleos(t)ide analogue therapy (Table 1) [6][8][38]. Moreover, indefinite combination therapy with HBIg and a NA may not be required in all liver transplant recipients. Both discontinuation of HBIg and/or NAs after a defined interval or continued treatment with antivirals alone [9][35][43][63] have been applied by some centers with promising results; these centers have achieved rate of recurrence between 0% and 12.9% (Table 1).

### 3.5. Hepatitis B Immunoglobulin Monotherapy

Some studies showed that HBIg monotherapy appeared to be equivalent to combination therapy (HBIg plus antiviral NA) for prevention of HBV post-LT as being more effective at reducing the re-infection rate [21][66]. Protocols for monotherapy with high doses of HBIg (10,000 IU) have been used during the anhepatic phase followed by daily dosing during the first few days post-transplantation, with subsequent continuation adapted according to serum anti-HB antibody titers to be either short term (6 to 12 months) or indefinite (Table 1) [4][5][14][15][19][28][30][31][32][34][35][36][37][38][41][47][48][52][57][67]. Long-term HBIg monotherapy is generally well tolerated, although mild to moderate adverse events have been noted [21][53]. However, due to the risk of late reinfection, especially in patients with active pre-LT replication of HBV (and particularly those with a viral load greater than 10(5) copies/mL), HBIg monoprophylaxis might not be a successful solution (Table 1) [4][11][15][20][68]. Furthermore, monotherapy with HBIg has been shown to promote mutations in the surface genes, which may lead to a reduction in the efficacy of HBIg [4][8][10]. Therefore, to overcome this problem, combination therapy of HBIg with NAs was introduced and has been accepted as being more effective at reducing the reinfection rate.

### 3.6. Combination Treatment of NAs With HBIg

The risk of recurrent hepatitis and death in transplant recipients has been lowered with the development of new antiviral agents, and the combined use of HBIg and these agents is associated with lower recurrence rates than HBIG or NA monotherapy. This prophylactic strategy includes potential concerted effects between HBIG and NA as well as shorter courses or lower doses of HBIG. In general, protocols for HBIg and antiviral NA combination therapy consist of pre-transplantation administration of antiviral agents (especially in HBV-DNA-positive patients) to reduce the viral load, followed by high doses of IV HBIg (10,000 IU/day) during the anhepatic phase, and a combination of long term antiviral NA and IV or IM HBIg following transplantation [21][69]. The use of combination therapy has become a common strategy to reduce post-LT HBV recurrence rates, and its efficacy has been investigated extensively [6][39][40][41][44][46][51][63]. Combination therapy appears to be an effective strategy for reducing HBV recurrence to 0 and 16% (Table 1) [6][39][40][41][44][46][51][63], and for bringing about >90% long-term negativity for HBsAg [70][71]. Further, combination therapy is more cost effective because the dosage of HBIg, which is expensive, can be reduced, making NA and HBIg combination therapy far more economically attractive and potentially more widely available [17][19][40][43][44][72][73][74][75][76]. Some studies have used NA alone in cases of post-LT HBV recurrence (on patient's demand) after administration of HBIg at doses currently defined as high and low in the anhepatic phase and post-LT (Table 1) [25][39][67]. However, combination protocols differ amongst transplantation centers and, to some extent, the reported results have been limited by small sample sizes and short-term follow-up. Nonetheless, the main problem with the use of antiviral substances before and/or after LT seems to be the evolution of resistant viral mutations, especially in high-risk patients [6][31][45][77][78]. New treatment options such as adefovir dipivoxil (ADF) and entecavir have been shown to prevent post-LT HBV infection [25], resulting in improved survival in HBV-infected transplant recipients [24][25][26].

### 3.7. New NAs

Among the new NAs, adefovir (ADF) has been evaluated in different studies as a treatment option in transplant settings [15][25][31][32][39][41][49][50][51][63] (Table 1). ADF therapy successfully suppressed HBV DNA replication and improved or stabilized HBV infection recurrence after LT [15][39]. LAM-resistant cases despite HBIg and lamivudine combination prophylaxis responded well to the addition of ADF to therapy or upon switching to ADF [4][25][32][39][63]. Some studies have used entecavir, both with and without HBIg, with promising outcomes and without any cases of HBV recurrence during the follow-up period [4][65][79][80][81][82]. A few studies on new NA drugs (ADF and entecavir) have also shown the effectiveness of these agents in the suppression of HBV replication in patients who developed lamivudine-resistance variants post-LT (Table 1). Although the use of these new antivirals for the prophylaxis of recurrent HBV infection is increasing without a concomitant increase in viral resistance, very few studies address the differences in the effect of the use of these new NAs as monotherapies or in combination with HBIg. Therefore, further studies are needed to test the effectiveness of these antivirals in combination therapies with HBIg and as monotherapeutic strategies [79][83][84].

## 4. Conclusions

Although a standard prophylactic regimen after LT is not available, the available systematic reviews and meta-analyses of clinical studies from multiple literature sources support the use of long-term combination therapy of HBIg and antiviral NAs (especially new agents).Regarding the combination strategy, the individualized prophylaxis protocols still are feasible, effective, but highly expensive. Most of the studies have a very small sample size that significantly limits the power of their findings. Future prospective multicenter and cohort studies with a longer period of follow up are needed to better define post-LT prophylactic strategies.
